# Clinical Trials of Oncolytic Viruses in Breast Cancer

**DOI:** 10.3389/fonc.2021.803050

**Published:** 2021-12-23

**Authors:** Mary E. Carter, André Koch, Ulrich M. Lauer, Andreas D. Hartkopf

**Affiliations:** ^1^ Department of Obstetrics and Gynaecology, University of Tuebingen, Tuebingen, Germany; ^2^ Department of Internal Medicine VIII, Medical Oncology & Pneumology, University of Tuebingen, Tuebingen, Germany

**Keywords:** oncolytic virus, virotherapy, breast cancer, clinical trials, review

## Abstract

Breast cancer is the second most common kind of cancer worldwide and oncolytic viruses may offer a new treatment approach. There are three different types of oncolytic viruses used in clinical trials; (i) oncolytic viruses with natural anti-neoplastic properties; (ii) oncolytic viruses designed for tumor-selective replication; (iii) oncolytic viruses modified to activate the immune system. Currently, fourteen different oncolytic viruses have been investigated in eighteen published clinical trials. These trials demonstrate that oncolytic viruses are well tolerated and safe for use in patients and display clinical activity. However, these trials mainly studied a small number of patients with different advanced tumors including some with breast cancer. Future trials should focus on breast cancer and investigate optimal routes of administration, occurrence of neutralizing antibodies, viral gene expression, combinations with other antineoplastic therapies, and identify subtypes that are particularly suitable for oncolytic virotherapy.

## 1 Introduction

One in eight women will be diagnosed with breast cancer in their life, and breast cancer is the most common kind of cancer in the United States. Around 90% of patients diagnosed with breast cancer and no sign of metastases survive the first five years and 86% survive the first 10 years (1, 2). Consequently, in comparison to other tumor entities, breast cancer has a relatively good prognosis. However, even decades after the primary diagnosis patients can still experience a relapse in the form of distant metastases. New therapeutic strategies such as cyclin-dependent kinase (CDK) 4/6 inhibitors, human epidermal growth factor receptor 2 (HER-2)-targeted therapy, or immunotherapy with checkpoint inhibitors can extend the survival when a patient is diagnosed with metastases (3–5). However, there is still no cure available (6, 7) and therefore a desperate need for new therapies.

Two of the main reasons for the development of a tumor are the combined changes in the genetic and epigenetic characteristics of a cell. These changes result in a higher probability of cells becoming immortal. Parallel to these changes, the evolving tumor cell produces neo-antigens which should cause the cell to be destroyed by the immune system. However, the cancer cell manages to circumvent the anti-tumor response by manipulating the body’s immune reaction. This effect is due to the decreased reaction to signals from the innate immune system, reduced expression of neo-antigens, and prevention of immune cells from infiltrating the tumor environment (8). These changes shield the tumor from the immune system but, interestingly, make it more vulnerable to the infection by viruses (9).

Oncolytic viruses represent a new approach to cancer treatment. In contrast to classic gene therapy, where replication incompetent viral vectors are used, oncolytic viruses are replication competent. Oncolytic viruses selectively infect tumor cells followed by proliferation of the viruses and destruction of infected cells, a process that is called oncolysis. The subsequent release of these additional infectious viruses causes the infection of neighbouring tumor cells (10, 11). At the same time a tumor-specific immune response is induced resulting in further enhancement of the oncolytic effects. Therefore, a combination of immunotherapies such as checkpoint inhibitors, that release the brakes on the immune system, with oncolytic viruses might be a promising therapeutic strategy; corresponding clinical trials are currently being undertaken (NCT02919449; NCT01937117).

The treatment of breast cancer with aggressive tumor biology such as triple-negative breast cancer (TNBC), defined by the absence of estrogen and progesterone receptors, as well as HER2, is challenging. Until recently, only chemotherapy was available to treat metastatic TNBC (12). A new therapeutic strategy, which has been shown to be effective in many solid tumors, is based on the inhibition of so-called immune checkpoints, specifically cytotoxic T-lymphocyte-associated antigen 4 (CTLA-4), programmed cell death -1 (PD-1), and programmed cell death-ligand-1 (PD-L1), which suppress the antitumor capabilities of the host immune system (13). Although historically breast cancer has been considered a non-immunogenic tumor (i.e. cold), TNBC appears more likely to respond to immunotherapy than other breast cancers because of an increased mutational burden, infiltration of the tumor microenvironment with immune cells (e.g., tumor-infiltrating lymphocytes), and higher expression of PD-L1 (14). Nevertheless, the efficacy of individual checkpoint inhibitors in TNBC is still low, and combinations of therapies are needed to overcome resistance to immunotherapy. Recently, combination with chemotherapy has been shown to be more successful (15). While some studies showed significant efficacy and led to approval of the checkpoint inhibitors pembrolizumab and atezolizumab in combination with chemotherapy for the treatment of metastatic and early TNBC, other studies have not been as successful (16–20). Although a long-lasting anti-tumor effect is seen in some patients, the vast majority of those treated do not respond to immunotherapy (21). Therefore, new strategies are needed to improve the efficacy of immunotherapy in breast cancer, which is not considered a highly immunogenic (i.e. hot) tumor compared to other entities such as melanoma or non-small cell lung cancer (22).

Novel treatment agents such as immune checkpoint inhibitors are an important milestone in response to the desperate search for novel therapeutic agents for breast cancer. However, there is still room to improve the clinical benefit for patients from this new treatment option (23). There are two potential ways of increasing the success. Firstly, subtypes of breast cancer susceptible to immune checkpoint inhibitors could be sensitized to improve the response to these therapeutic agents. Secondly, non-immunogenic tumors need to be transformed into immunogenic tumors thus making them more susceptible to immune checkpoint inhibitors. Oncolytic viruses may fulfil this role and offer a new way of improving treatment with immune checkpoint inhibitors. In particular, the activation of an immune response to the tumor cells due to viral infection may play an important part in this approach. In a Phase Ib clinical trial in advanced melanoma that combined the oncolytic virus talimogene laherparepvec with the anti-PD-1 antibody pembrolizumab researchers found that this combination enhanced the CD8^+^ T-cell count and elevated the PD-L1 protein expression. The authors suggest that this creates a changed tumor microenvironment, thereby potentially increasing response rates to an immune checkpoint inhibitor (24). Another study used a vesicular stomatitis virus (VSV) in combination with an anti-PD-1 checkpoint inhibitor as a therapeutic regime in experimental models of TNBC. The authors found that the recruitment of CD8^+^ T-cells plays an important role in enhancing the efficacy of immune checkpoint inhibitors (25). Additional laboratory studies underline the potential benefit of combining oncolytic viruses with immune checkpoint inhibitors (26, 27). The induction of an immune response to tumor cells, thereby sensitizing tumor cells to immune checkpoint inhibitors, enables oncolytic viruses to transform non-immunogenic tumors into more immunogenic tumors (28). Interestingly, because the transformation includes CD8^+^ T-cell recruitment, CD8-targeted positron emission tomography (PET) imaging may prove useful in future for evaluating oncolytic virotherapy in this context (29).

It is important to differentiate between oncolytic viruses with natural or intrinsic anti-neoplastic characteristics and oncolytic viruses that have been genetically modified (30). During the 20^th^ century wild-type oncolytic viruses were used and their effects on tumor cells were investigated. In the 1990s the next step was to genetically engineer these viruses for selective replication in tumor cells (31). The hope was to increase the oncolytic potential of such viruses. Herpes simplex virus type 1 (HSV-1) was the first oncolytic virus to be genetically modified by creating a thymidine kinase-negative mutant of HSV-1 (32). Many studies followed this development resulting in a variety of viruses which were found to exhibit tumor-selective replication (31, 33). More recently, an activated immune response against tumor cells caused by oncolytic viruses is understood to be important for their action (34–36). Importantly, a large randomized clinical trial phase III trial was undertaken using talimogene laherparepvec (T-VEC; Imlgygic^®^) in patients with advanced melanoma (37). A therapeutic benefit was shown (38).

**Figure 1:**
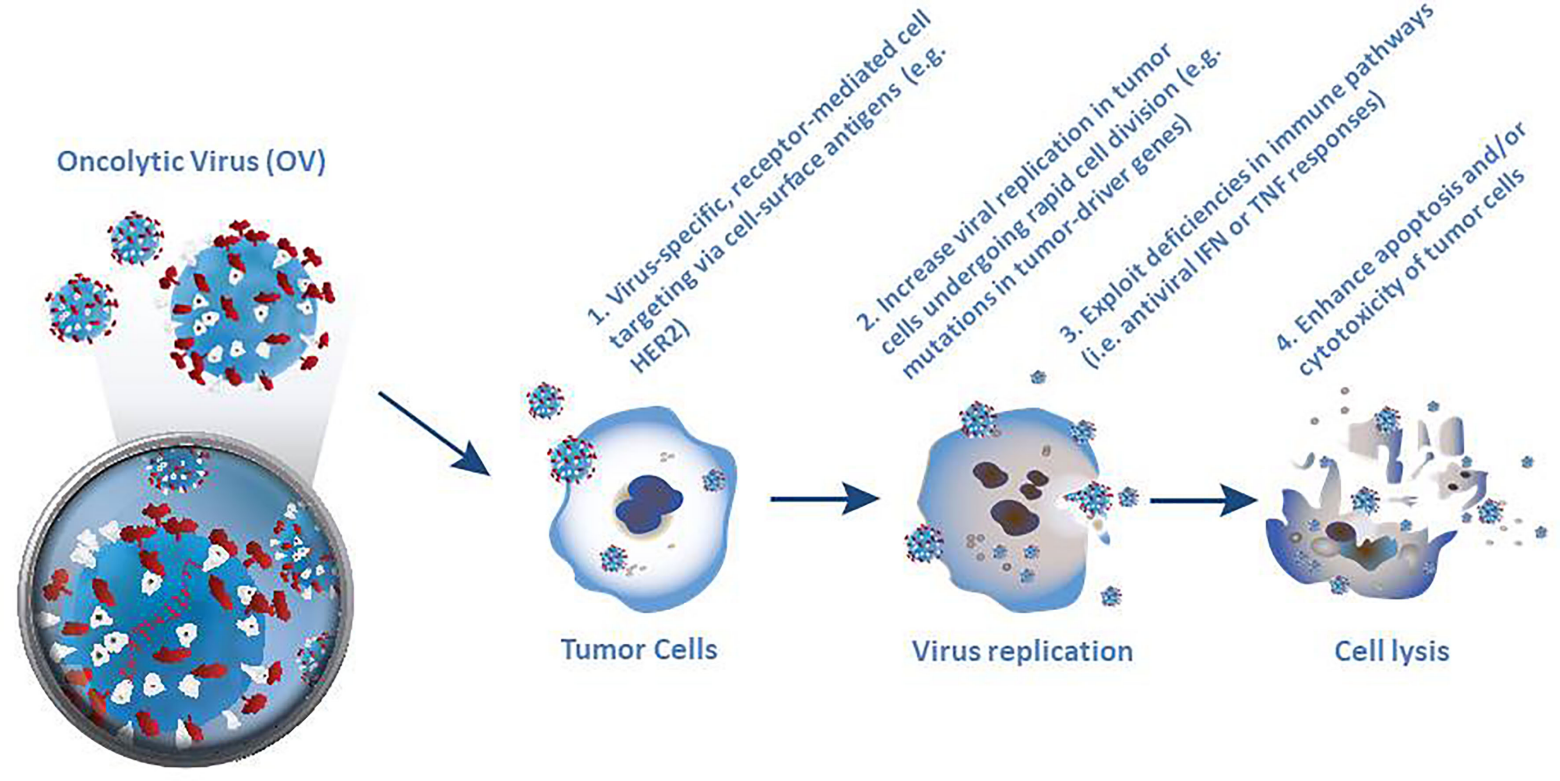
Four different ways used to engineer oncolytic viruses to selectively target tumor cells. Selective killing of tumor cells forms the first pillar of oncolytic virotherapy and requires specific targeting of cancer cells as a necessary pre-requisite for successful virotherapy. Although many naturally occurring viruses exhibit a natural preference for cancer cells, other viruses, need to be engineered to make them cancer specific.

Selective killing of tumor cells forms the first pillar of oncolytic virotherapy (**Figure 1**). Specific targeting of cancer cells is a necessary pre-requisite for successful virotherapy. Indeed, many naturally occurring viruses, such as parvovirus, measles virus, reovirus and Newcastle disease virus (NDV) exhibit a natural preference for cancer cells. However other viruses, such as adenovirus, VSV, vaccinia virus (VV) and HSV need to be engineered to make them cancer specific (30, 39). Four broadly different ways have been used to engineer oncolytic viruses to selectively target tumor cells. The first of these approaches utilizes virus-specific, receptor-mediated cell targeting based on addressing cell markers that are expressed in tumor cells, such as epidermal growth factor receptor (EGFR) and HER-2 (39). The second approach is based on the rapid cell division in tumor cells, which leads to high metabolic activity and replication rate, thereby supporting increased viral replication compared to normal quiescent cells (33). Moreover, mutations in tumor drivers or other enzymes such as protein kinase R (PKR) can increase the selectivity of virus replication in tumor cells (9, 36). Thirdly, many cancer cells exhibit deficiencies in normal antiviral interferon (IFN) or tumor necrosis factor (TNF) responses (30, 33) which encourage selective virus replication (39). Fourthly, normal cells respond to viral infection by inducing apoptosis or suppressing translational, transcriptional and/or transductional targeting to prevent the lysis of cells, which may limit the propagation of the virus (39).

**Figure 2:**
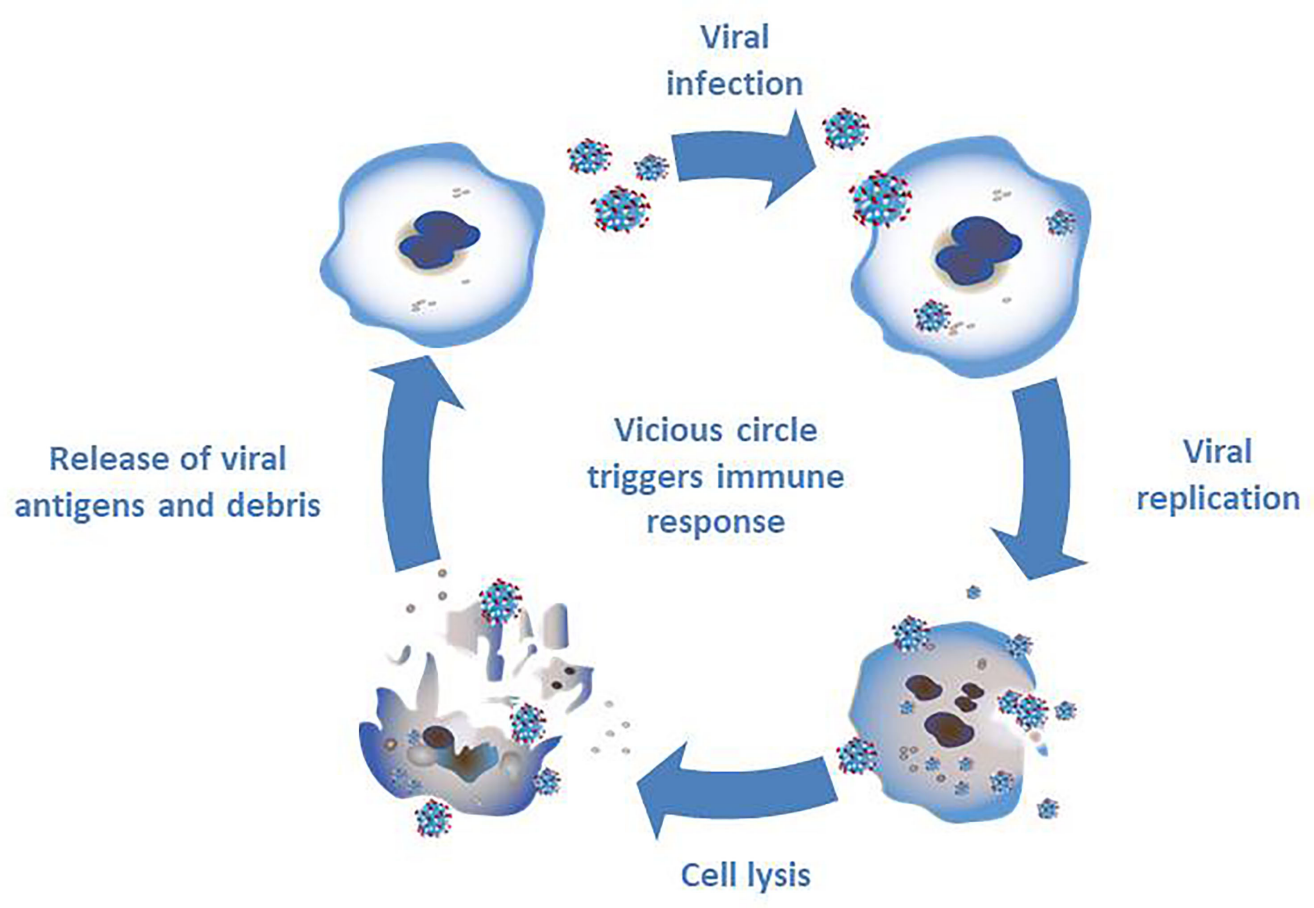
Triggering an immune response through infection with oncolytic viruses. The infection of tumor cells with oncolytic viruses results in viral replication and subsequent cell lysis. The debris and new antigens that are released through cell lysis result in a stimulation of the immune system.

The second pillar of oncolytic virotherapy is based on the immune response against tumor cells (**Figure 2**). The infection with oncolytic viruses results in a release of cell debris and antigens which stimulate the immune system (33). Normally the oncolytic virus would trigger an immune response in the cell leading to limitation of the viral infection. A combination of several factors - viral infection, oncolysis, new antigens and an activation of pathways normally signaling cell danger - may prevent the tumor in its microenvironment from evading the immune system and, thereby resulting in an immune response (33).

Oncolytic viruses are promising agents for the treatment of cancer because they selectively infect and destroy cancerous tissues without harming normal tissues (9). They also offer an attractive combination of tumor-specific cell lysis and immune stimulation. The first oncolytic virus approved in the US and EU was, talimogene laherparepvec, a genetically modified, live-attenuated, HSV-1-based vector, for the treatment of advanced melanoma, and unresectable metastatic melanoma respectively (38). Many more oncolytic viruses are currently being tested in clinical trials.

**Figure 3:**
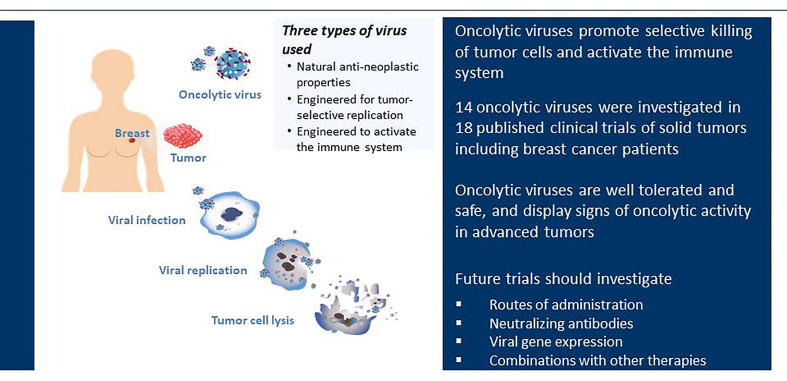
Clinical trials of oncolytic viruses in breast cancer. Oncolytic viruses selectively infect tumor tissue, undergo viral replication and cause tumor cell lysis. Currently, 14 different oncolytic viruses have been investigated in 18 published clinical trials. These oncolytic viruses fall into three different groups; (i) oncolytic viruses with natural anti-neoplastic properties; (ii) oncolytic viruses designed for tumor-selective replication; (iii) oncolytic viruses modified to activate the immune system. All published trials demonstrate that oncolytic viruses are well tolerated and safe for use in patients.

In this review, we highlight recent progress that has been made with oncolytic viruses specifically in the treatment of breast cancer with a focus on published clinical trials (**Figure 3** and **Table 1**). We have also searched unpublished clinical trials found on Clinical Trial Research: Trial Trove using oncology, breast as keywords for disease and lytic virus/virus, lytic as the keywords for therapeutic class.

**Table 1:** Published clinical trials with oncolytic viruses involving breast cancer patients.

Oncolytic virus	Modification	Type of study	Delivery/Combination	Ref.	Findings
**Vaccinia Virus**					
**Western Reserve strain JX-929/vvDD**	Deletion mutations of viral genes encoding VGF and TK	Phase I dose-escalation	Intratumoral injection in 16 patients with advanced solid tumors	(40)	No dose-limiting toxicity, selective infection of injected and non-injected tumors, antitumor activity.
**Reovirus**					
**Pelareorep (Reolysin^®^)**	Purified live replication-competent form of reovirus serotype 3 Dearing strain	Phase I dose-escalation	Intravenous administration in 18 patients	(41)	Safe and well tolerated. All patients developed neutralizing antibodies, 6 exhibited viral shedding.
		Phase I dose-escalation	Intratumoral injection in 19 patients with advanced tumors	(42)	Safe and well tolerated, (local erythema and flu-like symptoms. Tumor response in 7/19 patients.
		Randomized Phase II safety and efficacy	Intravenous administration with paclitaxel, 74 patients with metastatic breast cancer	(43)	Well tolerated, no difference in the primary endpoint of PFS, but overall survival was prolonged by combination.
**Adenovirus**					
**Ad5/3-D24-GMCSF (CGTG-102)**	Serotype 5/3 capsid-modified adenovirus encoding GMCSF	Phase I	Single/subsequent intratumoral injection, 115 patients solid tumors	(44)	Well tolerated. Correlation between antiviral and anti-tumor T cells observed.
		Phase I in combination with cyclo-phosphamide	Intratumoral and/or intravenous or intraperitoneally in combination with oral or intravenous cyclophosphamide in 16 patients	(45)	Well tolerated. Co-treatment with cyclophosphamide showed possible antitumor activity with evidence of tumor shrinkage in 3 of 14 imaged patients
**Ad5/3-E2F-Δ24-GMCSF (CGTG-602)**	Tumor-specific E2F1 promoter for enhanced tumor selectivity and GMCSF	Phase I	Intratumoral injection in 13 patients	(46)	Well tolerated, frequent tumor- and adenovirus-specific T-cell immune responses. Efficacy seen in 9/12 evaluable patients based on tumor marker or radiological responses.
**Ad5-D24-GMCSF**	Serotype 5 adenovirus encoding GMCSF	Phase I dose escalation	Intratumoral injection in 20 patients advanced solid tumors	(47)	Well tolerated with induction of tumor-specific adenovirus virus-specific immunity. Evidence of clinical response
		Phase I combination with cyclophosphamide	Intratumoral injection of adenovirus with intravenous and/or oral cyclophosphamide	(48)	Well tolerated. Co-treatment with cyclophosphamide resulted in higher disease control than virus alone
**Ad5-RGD-D24, Ad5-RGD-D24-GMCSF**	RGD-4C modification for viral entry and GMCSF	Phase I	Intratumoral/intraperitoneal and intravenous injection, 16 patients with solid tumors	(49)	Well tolerated, 10/13 measurable viral circulation after 2 weeks, evidence of disease stabilization in some patients
**ICOVIR-7**	RGD-4C modification and 24-bp deletion in the E1 region conferring cancer cell specificity	Phase I dose-escalation	Intratumoral injection, 21 patients with various advanced metastatic solid tumors (3 breast cancer patients)	(50)	Tolerated, neutralizing antibody titre induced in 4 weeks in 16/18 patients, viral genomes were detected in 18/21 patients and 7/15 patients were still positive 2-4 weeks later. Antitumor activity seen in 9/17 evaluable patients.
**Telomelysin**	hTERT promoter and replacement of transcriptional element of viral E1B gene by an IRES sequence	Phase I	Single intratumoral injection, 16 patients with solid tumors	(51)	Well tolerated with injection site reactions and fever/chills. hTERT expression in 9/12 patients and viral DNA in 13/16 patients. 7 patients with stable disease 56 days after treatment
**H103**	Overexpression of Hsp70	Phase I, dose-escalation	Intratumoral injection, 27 patients with various advanced solid tumors	(52)	2 patients developed dose-limiting toxicities (fever and thrombocytopenia), otherwise mild to moderate. 3/27 patients complete/partial responses.
**ONYX-015, dl-1520, lontucirev**	Deletion of E1B-55K and E3B regions	Phase I does-escalation	Intravenous infusion in combination with etanercept, 9 patients with various advanced solid tumors	(53)	No significant adverse events attributed to the experimental regimen. 2/3 patients had detectable viral DNA at days 3 and 8 post-ONYX-015 infusion. 4/9 patients showed stable disease.
**Newcastle Disease Virus**					
**PV701**	Purified, naturally attenuated, replication competent isolate	Phase I	Intravenous injection of single/repeated doses, 79 patients with various advanced solid tumors	(54)	Flu-like symptoms commonest side effect together with injection site reactions. Desensitization to adverse events with subsequent doses.
		Phase I dose- escalation	Intravenous injection, two-step desensitization, 16 patients with advanced solid tumors	(55)	No dose-limiting toxicities, mild flu-like symptoms diminished with repeated dosing. 1 patient partial response, 4 patients with disease stabilization.
**Herpes Simplex Virus**					
**HF10**	Mutant, incomplete UL56 gene product	Phase I	Intratumoral injection, 6 patients with recurrent metastatic breast cancer	(56–58)	Well tolerated, no adverse events. Possible tumor regression and infiltration of CD8+ and CD4+ T cells
**OncoVEX^GM-CSF^**	Deletion of ICP34.5 and ICP-47, and insertion of GMCSF	Phase I	Intratumoral injection of single and multiple doses, 30 patients with metastases from solid tumors (14 breast)	(59)	Well tolerated, local inflammation, erythema, febrile responses. Virus replication, local reactions, GMCSF expression, and HSV-associated tumor necrosis. Some histopathological anti-tumor effects.

GM-CSF, Granulocyte-macrophage colony-stimulating factor; hTERT, human telomerase reverse transcriptase gene; IRES, Internal Ribosomal Entry Site; Hsp70, Heat shock protein 70; VGF, vaccinia growth factor; TK, thymidine kinase; ICP, infected cell protein; PFS, progression-free survival.

## 2 Published Clinical Trials With Oncolytic Viruses in Breast Cancer

There have been several previous publications that have listed oncolytic viruses in ongoing or completed trials, but none of these provide a systematic review of the published clinical trials (60–63). Our review provides for the first time an in-depth systematic review of all published clinical trials with oncolytic viruses that included breast cancer patients.

In total, 14 different oncolytic viruses have been tested in 18 published clinical trials for the potential treatment of breast cancer as of 26 November 2021 (**Table 1**). **S1 Supplementary Information** provides more details on the mechanisms of the used oncolytic viruses used in the clinical trials and the results of these studies. As of 17 April 2021, there were 62 ongoing clinical trials with oncolytic viruses in patients with breast cancer (**S2 Supplementary Information**). The search found another 2 clinical trials with therapeutic agents not classified as oncolytic viruses.

We have divided the oncolytic viral agents into three different sections for the purpose of our review. The first section describes oncolytic viruses with natural anti-neoplastic properties. The second section focuses on oncolytic viruses that are designed for tumor-selective replication. The third section describes the clinical trials with oncolytic viruses genetically modified to activate the immune system (armed oncolytic viruses).

### 2.1 Oncolytic Virus With Natural Anti-Neoplastic Properties

#### 2.1.1 Newcastle Disease Virus

Newcastle disease virus (NDV) causes Newcastle disease (also called Ranikhet disease) and is characterized by a single-stranded, negative sense, non-segmented RNA with the diameter reaching 200-300 nm. It possesses a pleomorphic envelope and the genome encodes seven essential genes, namely nucleocapsid protein, phosphoprotein, matrix protein, fusion protein, haemagglutinin-neuraminidase (HN) protein, the RNA-dependent RNA polymerase, and the V protein (64, 65).

In birds, many different avian species and manifestations of NDV have been reported (65). NDV can be differentiated into three categories. The first category is characterized by a low virulence and is therefore called lentogenic. The second category exhibiting moderate or intermediate virulence is named mesogenic. Finally, the third category of NDV is velogenic and is characterized by high virulence and is further classified according to its predilection site, i.e. whether it is neurotropic or viscerotropic (66). Only the mesogenic and the velogenic pathotype exhibit an oncolytic potential (64).

NDV is a paramyxovirus (65) and innately grows in cells with deficient interferon (IFN) signaling, like many tumor cells (67). The V protein is essential for the interaction and inhibition of IFN, thereby resulting in an increased virulence. The HN protein increases the apoptosis rate in infected tumor cells (65). PV701 is a naturally occurring NDV (55) which is both velogenic and lytic (65). It originates from an avian paramyxovirus thereby making it suitable for use in humans. A Phase I trial concluded that adverse effects (flu-like symptoms) were common and of higher intensity after the first application of PV701 (54). A following Phase I clinical trial tested the effect of a two-step desensitization with intravenous administration of PV701 on the side effects (55). Sixteen patients with incurable solid tumors including two patients with breast cancer were eligible. The treatment scheme included two cycles of six PV701 applications each over a period 15 days with a subsequent 6-day rest. The first dose was lower than the following ones. As a result, the maximum tolerated dose was not reached. The most commonly observed adverse reactions were flu-like symptoms. The intensity of these symptoms decreased with each additional application of PV701. One patient showed a partial response and four patients with progressive disease at the time of enrolment displayed disease stabilization ≥ 6 months (55).

#### 2.1.2 Pelareorep

Pelareorep (Reolysin) is another naturally occurring oncolytic virus. Pelareorep originates from a double-stranded RNA reovirus serotype 3 Dearing strain (41). The term reovirus (respiratory enteric orphan virus) was used to describe a group of cytopathogenic viruses that have three distinct human serotypes and cause mild gastrointestinal or upper respiratory infections in humans (68). The inhibition of the cellular double-stranded RNA-activated protein kinase in tumor cells with an activated RAS-pathway causes tumor selective oncolysis (41). Moreover, there is evidence that EGFR mutations facilitate infections even of tumor cells without activated RAS (69). In a Phase I trial of patients who suffered from advanced or metastatic solid cancer that did not respond to current available treatment, different doses of pelareorep were applied intravenously every four weeks. Eighteen patients were eligible including two patients with breast cancer. No maximum tolerated dose was found and treatment was well tolerated (one patient experienced fatigue and another patient fever). One patient displayed a partial response (anthracycline and taxane refractory breast cancer), showing necrosis and viral shedding in a biopsy taken from her chest wall. The clinical benefit rate was 45%. As effectivity was higher in patients with viral shedding (in serum, saliva, stool, or urine) this may be indicative of a higher replicative activity in these patients, thereby paving the way to clinical response (41). A subsequent Phase I study investigated escalating intratumoral doses of pelareorep in patients with advanced tumors including three with breast cancer. Pelareorep was well tolerated in this study with only local grade 2 erythema and flu-like symptoms observed. There was some evidence of local target tumor response activity in 7 of 19 patients with one breast cancer patient exhibiting stable disease after six or more weeks (42).

The combination of pelareorep and paclitaxel to treat metastatic breast cancer was evaluated in a multicenter randomized Phase II trial. A total of 81 patients that had received chemotherapy for advanced disease were enrolled in the study. Seven patients were part of a safety-run. The remaining 74 patients either received a combination of paclitaxel and pelareorep (n=36) or paclitaxel mono-treatment (n=38). The primary endpoint was progression-free survival, and secondary endpoints included objective response rates, overall survival, circulating tumor cell counts and safety. Pelareorep was well tolerated. After a median follow-up of 29.5 months, progression-free survival was 3.78 months in the combination arm and therefore not significantly different as compared to 3.38 months (HR 0.8; 80% CI 0.54–2.22; p=0.87) for paclitaxel alone. Although there was also no difference in response rates, median overall survival was slightly, but not significantly better: 17.4 for the combination versus 10.4 months (HR 0.65; 80% CI 0.46–0.91; p=0.1) for paclitaxel alone (43). Currently, there are 14 ongoing clinical trials using pelareorep to treat metastatic breast cancer including three Phase III trials (S2).

#### 2.1.3 HF10

Herpes simplex Virus (HSV) is a double-stranded DNA virus that causes a variety of diseases ranging from mild skin disorders to fatal encephalitis. HF10 is a spontaneously occurring oncolytic mutant of HSV-1 with a unique genomic structure that has non-engineered genetic deletions and insertions (70). The genomic alterations result in an incomplete UL56 gene product thereby leading to a reduced capability to invade the central nervous system which enhances safety significantly. Six patients diagnosed with breast cancer with > 10 metastases at cutaneous or subcutaneous sites were enrolled in a Phase I clinical trial. HF10 was injected into one tumor nodule and saline solution was injected into a different nodule of the same patient once daily over a period of three days. After 14 days the nodules were removed for histopathologic examination. The application of HF 10 was well tolerated. Histopathological evaluation showed that cell death occurred in 30 to 100% of malignant cells in patients injected with HF10, whereas no cell death was observed in the saline-injected nodules (56).

### 2.2 Oncolytic Viruses Designed for Tumor-Selective Replication

#### 2.2.1 Vaccinia Virus

Vaccinia virus (VV) is a linear, double-stranded DNA virus belonging to the genus *Orthopoxvirus* of the family *Poxviridae* (71). Typically, VV infection produces four different virions that have different abundance, structure, location and roles in the virus life-cycle: (i) the intracellular mature virus (IMV), (ii) extracellular enveloped virus (EEV), (iii) intracellular enveloped virus (IEV), and (iv) the cell-associated enveloped virus (CEV) (71). Cell lysis results in the release of large numbers of IMV which are more stable than EEV and easily detected by the immune system. The additional membrane of EEV originating from the host cell results in enhanced immune evasion and a greater spread (72). JX-929 (vvDD) is a genetically engineered Western Reserve strain VV with two gene deletions (40, 73). Through the deletion of the thymidine kinase gene the viral DNA synthesis is dependent on thymidine triphosphate from dividing cells such as tumor cells. The deletion of the vaccinia growth factor (VGF) gene stops neighbouring cells from dividing (74). JX-929 includes a homologous recombination of the cytosine deaminase that enables infected cells to convert 5-flurocytosin to 5-flurouracil (75). It also includes the somatostatin receptor for imaging of viral spread through imaging the accumulation of radioactivity of ^111^In-pentetreoide in infected cells (73). Sixteen patients with tumors unresponsive to current treatments were enrolled in a Phase I clinical trial including three with breast cancer (40). The virus was injected directly into the tumor. No dose-limiting toxicity was found at the doses given in this clinical trial. The adverse effects displayed included fever, malaise and pain. These symptoms correlated with the replication of vvDD and the immune response against vvDD. Thus, Western Reserve strain oncolytic VV appears safe for use in patients and shows selective replication in injected and un-injected tumors.

#### 2.2.2 Adenovirus

Adenoviruses (Ad) contain double-stranded linear DNA of 38 kB. Over the years more than 40 different serotypes have been discovered in humans of which serotypes 2 and 5 are the ones currently mostly being used as oncolytic adenoviruses (76). Adenoviruses express certain proteins that result in an evasion of the immune system. Additionally, the suppression of apoptosis through adenoviruses is due to proteins interacting with Fas ligand and TNF pathways. Three main mechanisms have been shown to result in cell death after adenovirus infection. Firstly, adenoviruses can directly cause cytotoxicity. Secondly, they can alter the immune response to the tumor cells through increased sensitivity to cytokines or induction of cytokine production (i.e. TNF). Thirdly, adenoviruses can increase the response to chemotherapies (77). Oncolytic adenoviruses can be classified into two groups. The first group consists of adenoviruses in which genes have been modified to reduce replication and infection in normal cells. The second group includes adenoviruses that have been modified to specifically target cancer cells (78). Currently, there are 13 ongoing clinical trials investigating adenoviruses for the treatment of breast cancer (S2).

#### 2.2.3 ICOVIR-7

ICOVIR-7 is an adenovirus which has been genetically altered, including a deletion allowing the regulation of a gene by a tumor-specific promoter E2F-1 (79). E2F-1 regulates parts of the Rb-p16 pathway which is defective in many tumor cells (50). Moreover, this modification includes a change to the serotype 5 adenovirus with the aim of facilitating specific entry into tumor cells and improving the infection of cancerous cells. Further modifications were made to enhance transcription. A clinical trial evaluated the effects of ICOVIR 7 in patients with advanced solid tumors. A total of 21 patients were enrolled including three with breast cancer. ICOVIR 7 was applied once intratumorally in different doses. The side effects included fever, fatigue, elevated liver transaminases, chills and hyponatremia. Grade 3 anemia was diagnosed in one patient. Viral replication was indicated by circulating viral DNA in 18 patients and in 7 tissue samples 2 to 4 weeks after treatment. After treatment two stable diseases, two minor responses and one partial response occurred in 12 patients with available follow-up data. One of the patients with breast cancer exhibited a decrease or stabilization of tumor markers (50).

#### 2.2.4 Telomelysin

Telomelysin (OBP-301) is another genetically modified adenovirus and is based on the serotype 5 adenovirus. This oncolytic virus includes a promoter for the human telomerase reverse transcript gene (hTERT) (51), which is responsible for maintaining the lengths of telomeres at the end of chromosomes. An upregulation in the telomerase pathway is seen in many cancer cells (80). A total of 16 patients were enrolled in a Phase I trial of patients with advanced solid tumors of which only one had breast cancer. Telomelysin was applied once intratumorally at three different doses. The application of telomelysin was well tolerated and only mild adverse effects were seen (such as pain and induration at the site of injection as well as fever and chills). Post-treatment biopsies of the tumors showed hTERT expression in 9 of 12 patients, thereby indicating a permissiveness of these tumors to support viral replication. One partial response and 7 stable diseases occurred at a follow-up 56 days after treatment (51).

#### 2.2.5 ONYX-015/dl1520

The adenovirus ONYX-015/dl1520 (lontucirev) belongs to the group C of adenoviruses. A genetic modification causes a deletion of the E1B-55K and E3B region. Previous clinical trials using ONYX-015 mono-therapy rarely displayed a clinical benefit for patients although biological activity was seen (81). Research has shown that TNF-α is one of the most important cytokines in the immune response towards adenoviruses and an important pro-apoptotic factor (82, 83). Different regions in the genome of adenoviruses encode proteins to minimize the negative effect TNF-α has on cells infected through adenoviruses (84). Therefore, the deletion of these regions helps to induce apoptosis in cancerous cells when infected with this virus. Another region contains a protein that interferes with antigen presentation. Its deletion facilitates an immune response directed at infected tumor cells (85).

A Phase I trial investigated the combination of ONYX-015 together with the synthetic dimer of the human TNF-α receptor etanercept (53) in patients with solid tumors including two patients with breast cancer. Only mild side effects were seen. All patients developed mild to moderate fever 24 h after treatment with ONXY-015 and in some patients, hyponatremia and transient transaminitis were also seen. Two of the three patients that received the highest dose of ONYX-015 expressed measurable viral DNA. The quantity of the viral DNA was higher in cycle 1 than in cycle 2. As etanercept was only administrated in cycle 1, the reduced quantity could be attributed to the lack of etanercept in cycle 2. Overall, 4 of 9 patients showed a stable disease (53). A study with ONYX-015 concluded that heat shock proteins facilitate the export of viral mRNA necessary for an efficient infection of cancerous cells (86).

### 2.3 Armed Oncolytic Viruses

#### 2.3.1 Serotype 5/3 Adenovirus

There are several examples of armed oncolytic adenoviruses in clinical trials that included breast cancer patients. The serotype 5/3 adenovirus has been modified to express the granulocyte-macrophage colony-stimulating factor (GMCSF) by creating the constructs Ad5/3-E2F-Δ24-GMCSF (CGTG-602) and Ad5/3-D24-GMCSF (CGTG-102) (44, 46). GMCSF has been shown to activate antigen-presenting cells with the majority being dendritic cells. Additionally, the innate immune system is activated including a sequestration of natural killer cells and neutrophils (46). A clinical trial using CGTG-602 enrolled thirteen patients with metastatic tumors including three with breast cancer (46). The adenovirus was administrated intratumorally (3 injections per patient). At least stable disease was seen in 83% of the patients. Response rate as demonstrated by positron emission tomography (including minor metabolic response) was 50% including one breast cancer patient. Post-treatment biopsies indicated an active immune response towards the tumor through increased number of infiltrating immune cells such as T cells. Additional RNA expression analyses of these biopsies suggested metabolic changes due to viral infection (46). In a subsequent study, the induction of antitumor immunity was studied in patients with solid tumors (16 with breast cancer) by comparing a single intratumoral injection of Ad5/3-D24-GMCSF (CGTG-102) with the administration of three subsequent doses 3 to 4 weeks apart (44). The results of this study provided the first data linking antiviral immunity with antitumor immunity. No analysis of efficacy according to tumor subtypes was performed in this study. In addition, a combination of Ad5/3-D24-GMCSF and low-dose cyclophosphamide was administered to 16 patients with advanced breast cancer and found to be well tolerated with evidence of tumor shrinkage in 3 of 14 imaged patients (45).

#### 2.3.2 Serotype 5 Adenovirus

The serotype 5 adenovirus has also been engineered to express human GMCSF (Ad5-D24-GMCSF) and used to treat patients with advanced solid tumors including two patients with breast cancer. The results showed that intratumoral injections of Ad5-D24-GMCSF were well tolerated and clinical responses were frequently seen. One breast cancer patient exhibited disease stabilization and the second demonstrated a reduction of tumor markers to normal. Interestingly, this study also showed evidence of both tumor-specific and virus-specific immunity (47). Another clinical trial used the two adenoviruses Ad5-RGD-D24 and the GMCSF-encoding variant Ad5-RGD-D24-GMCSF. These viruses have been modified with arginine (R)–glycine (G)–aspartic acid (D) (RGD) - targeting integrin, which allows cell entry *via* alpha-v-beta-integrins often expressed in tumor cells. Moreover, GMCSF controlled by an E3 promoter was included into Ad5-RGD-D24-GMCSF (49). To further enhance cancer selectivity a 24 base-pair deletion was introduced into the region 2 of E1A to induce a cytostatic effect (49, 87). In a Phase I trial using Ad5-RGD-D24 and Ad5-RGD-D24-GMCSF 16 patients with solid tumors including breast cancer were enrolled (49). Nine patients were treated with Ad5-RGD-D24, of which two had breast cancer, and 7 patients were treated with Ad5-RGD-D24-GMCSF, of which none had breast cancer. One fifth of the dose was given intravenously and four fifths were given intratumorally. Generally, the application was well tolerated but typical side effects included mild to moderate fatigue, fever and pain at the site of injection. Ten of 13 patients with available data showed measurable viral circulation two weeks after treatment. Half of the patients treated with Ad5-RGD-D24-GMCSF showed a stable disease after one application of the virus. Additionally, two thirds of patients treated with Ad5-RGD-D24-GMCSF displayed stabilized or reduced tumor marker levels. In contrast, all patients treated with Ad5-RGD-D24 showed disease progression, while half of these patients had temporary improvements of tumor marker levels. Ad5-RGD-D24-GMCSF treated patients exhibited signs of an immune response directed towards the tumor and virus (49). Finally, a study was published that looked at the immunological effects of low doses of the alkylating agent cyclophosphamide in patients treated with the oncolytic adenoviruses Ad5-D24-GMCSF, Ad5/3-D24-GMCSF, Ad5-RGD-D24-GMCSF and ICOVIR-7 (48). A total of 43 patients with advanced solid tumors including 3 with breast cancer received intratumoral injections of one of the adenoviruses and some of these also received infusions and/or oral low-dose cyclophosphamide. All treatments were well tolerated. Antibody formation and virus replication were not affected by the administration of cyclophosphamide. Interestingly, oncolytic adenovirus administered together with metronomic cyclophosphamide (i.e. oral or oral plus intravenous regimens) increased cytotoxic T cells and induced Th1 type immunity in patients. All cyclophosphamide regimens resulted in higher rates of disease control compared to virus alone, although it was not possible to determine the specific affects in any of the three breast cancer patients or which patients received which particular adenovirus (48).

#### 2.3.3 H103

H103 is a recombinant oncolytic serotype 2 adenovirus overexpressing heat shock protein 70 (Hsp70) (52). During oncolysis Hsp70 is released from infected tumor cells. These proteins then act as an epitope for antigens thereby stimulating a systemic immune response (52, 88). A study using a Hsp70-mediated cancer tumor vaccine resulted in a decrease of tumor sizes and metastasis (88). Therefore, agents or mutations that lead to an increase of heat shock proteins may result in an improved therapeutic response (86). A Phase I clinical trial using an oncolytic virus H103 expressing Hsp70 was initiated with 27 patients with solid tumors non-responsive to current available treatment options, of which one patient had breast cancer amongst six with other tumors (52). The virus was applied intratumorally as a single or multi-dose application. Mainly mild adverse events were seen such as fever, pain at the site of injection and a local reaction. Two patients showed severe fever and transient thrombocytopenia. Three patients showed a partial or complete response in the original tumor and another three patients also displayed response in metastases not injected with the oncolytic virus. It did not appear that the single breast cancer patients exhibited any treatment-related effects in this study (52).

#### 2.3.4 OncoVEX^GM-CSF^

OncoVEX^GM-CSF^ (other names are T-VEC and talimogene laherparepvec (Imlgygic^®^)) is a recombinant herpes simplex virus type 1 (HSV-1). It is a JS1 strain of HSV-1 with a deletion of ICP34.5 and ICP47 (59). A mutation in the neuro-virulence factor ICP34.5 enables greater cell killing potential selective for tumors (89). PKR is activated through stress signals such as viral infections. It results in a halting of mRNA translation of viral or cellular origin. PKR is also capable of inducing apoptosis (90). The deletion of ICP47 induces an activation of the immune system resulting in an immune response not just against the virus but also against infected cells. The systemic immune response is further intensified through the expression of the GMCSF gene which is encoded in this virus as a therapeutic transgene (38). The CMV promoter regulates the GMCSF gene and was added into the OncoVEX^GM-CSF^ genome in the place of ICP34.5. As HSV infections and sero-positivity are common in the population it is important to note that an effective tumor treatment with OncoVEX^GM-CSF^ is still achievable when applied intratumorally; as a result of this route of application any local preformed anti-HSV-1 immune response is overwhelmed by the huge number of locally applied infectious viral particles (up to 10^8^ OncoVEX^GM-CSF^ particles). Clinical studies conducted in melanoma patients indicated that there is a response to OncoVEX^GM-CSF^ not just in injected lesions but also in distant metastases, defining a so-called abscopal effect (37, 91). Talimogene laherparevac (T-Vec) expresses GM-CSF thereby stimulating cytokine production and potentially activating the immune response, and perhaps offering itself to combinations with immune checkpoint inhibitors (92).

A Phase I clinical study of patients with advanced solid tumors included 13 patients in a single-dose group and 17 patients in a multi-dose group, of which 26 patients could be evaluated (59). Of the 30 patients enrolled in the study, 14 patients had breast cancer and 13 of these could be evaluated. Only mild side effects such as local symptoms (i.e. inflammation and erythema) and fever were seen. HSV antigen-associated tumor necrosis indicated viral replication. After treatment three patients showed stable disease, including one patient with breast cancer, six patients experienced a decrease of tumor size (injected and/or un-injected), including two with breast cancer, and four patients displayed additional inflammation in un-injected lesions. Overall, the multi-dose regime seemed more promising than single-dose application (59). OncoVEX^GM-CSF^ was approved by the United States Food and Drug Administration (FDA) in 2015 for the treatment of melanoma, specifically for patients with lesions which are not accessible occurring after initial surgery. The European Medicines Agency’s (EMA) Committee for Medical Products for Human Use also approved OncoVEX^GM-CSF^ in 2015 for unresectable melanoma with certain types of metastasis (38). Currently, there are nine ongoing clinical trial using OncoVEX ^GM-CSF^ (S2).

## 3 Conclusions

We have reviewed the different viruses that have been investigated in published clinical trials for the treatment of breast cancer (**Figure 1** and **Table 1**). We have focused primarily on clinical trials for which results have been published in peer-reviewed journals. Although oncolytic viruses have been proposed as a future treatment option for the treatment of cancer for several years, the results of the clinical trials published so far demonstrate mixed results. There are currently 18 published clinical trials (17 Phase I studies and 1 Phase II study) with 14 different oncolytic viruses from five different viral families (VV, reovirus, adenovirus, NDV, HSV). We found that the adenovirus was the most common oncolytic virus tested in clinical studies that included patients with breast cancer (10 of 18 clinical trials). This corresponds well with a recent review describing the overall clinical landscape for oncolytic viruses in all types of cancer which highlighted that the adenovirus was the most common virus type tested in all clinical trials for cancer (93).

The oncolytic viruses used in the published clinical trials can be grouped into (i) wildtype/natural mutant viruses, (ii) viruses genetically engineered for tumor-selective replication, and (iii) viruses genetically modified to activate the immune system. The first group includes the three following viruses: pelareorep, PV701 and HF10) (41–43, 54–57). The second group includes four oncolytic viruses, namely JX-929, ICOVIR-7, telomelysin and ONYX-015). The third group includes the following seven oncolytic viruses, namely the adenoviruses Ad5/3-D24-GMCSF (CGTG-102), Ad5/3-E2F-Δ24-GMCSF (CGTG-602), Ad5-RGD-D24, Ad5-RGD-D24-GMCSF, Ad5-D24-GMCSF, H103, and talimogene laherparepvec (44, 46–49, 52, 59). The last group also includes the only oncolytic virus talimogene laherparepvec (Imlygic^®^) which has been licensed by both FDA and EMA for advanced stages of melanoma (38). Based on the available published clinical trials adenoviruses (double-stranded DNA virus) seem very promising because the majority of clinical trials that include breast cancer patients have been performed with this type of virus. Additionally, there has been broad research into genetic engineering of adenoviruses to activate the immune system. This makes them an ideal combination partner for immune checkpoint inhibitors. However, pelareorep (double-stranded RNA virus) appears very promising as this virus was the only one to be tested in a published Phase II clinical trial in this review. There are currently, 14 ongoing clinical trials with pelareorep including breast cancer patients (see **S2 Supplementary Information**). Indeed, only time will tell whether these oncolytic viruses prove the most promising, especially in combination with arising immunotherapies such as immune checkpoint inhibitors.

Overall, the results of the studies show that most oncolytic viruses were found to be safe and well tolerated with few side effects mostly limited to flu-like symptoms or local inflammation at the injection sites. One study demonstrated local reactions that dissipated with repeated dosing (54). There did not appear to be any great differences between the various oncolytic viruses with respect to tolerability with possibly the exception of H103 where 2 patients developed high-grade fever and thrombocytopenia (52).

The published reports described two different routes of administration. Eleven of the studies reported intratumoral administrations of the oncolytic virus (40, 42, 44, 46–48, 50–52, 56, 57, 59), five described intravenous injections (41, 43, 53–55), and one study used both routes of administration (49). This indicates that the best routes of administration have not been defined so far.

Four clinical studies attempted to determine whether treatment induced antibodies to the oncolytic virus and found that they could identify neutralizing anti-viral antibodies (41, 42, 48, 50). It remains to be seen what effect such anti-viral antibodies will have on subsequent treatments with the same oncolytic virus. Five trials also documented T cell responses directed to the oncolytic viruses (44, 46–48, 56, 57). Finally, only two studies demonstrated viral gene expression (51, 59), whilst seven studies measured viral shedding/DNA after treatment (41, 42, 44, 49–51, 53). Ideally, it would help virologists if parameters such as viral gene expression, viral shedding and neutralizing antibodies could be measured in a more systematic pattern as more clinical trials are performed. This will help to understand dosing and responses of the oncolytic viruses to a greater degree. Furthermore, if the field expands more towards armed oncolytic viruses that activate the immune system, future trials will need to measure the effects on the body’s innate and adaptive immune responses and potentially combine such approaches with other cancer immunotherapies.

Finally, most of the published clinical trials investigated the effects of oncolytic viruses in patients with advanced metastatic solid tumors of which only some were breast cancers. The studies were all Phase I trials except for one. Thus, it is very difficult to draw any reliable conclusions about efficacy especially with regard to breast cancer patients. Indeed, the only published randomized Phase II trial designed to show efficacy in breast cancer was one involving intravenous administration of the reovirus pelareorep in combination with paclitaxel (43). Regrettably, this study failed to show a significant difference in the primary endpoint of progression-free survival. We are therefore only at the beginning of our clinical journey with oncolytic viruses. Hopefully, many of the 62 ongoing clinical trials in breast cancer that have not yet been published will provide more information about ideal routes of administration, neutralizing anti-viral antibodies, viral shedding and combinations with immunotherapy and most importantly therapeutic effectiveness.

Finally, we have no evidence from these trials whether particular subtypes of breast cancer are particularly suitable for oncolytic virotherapy and at which stages virotherapy could be applied with an optimal outcome. Patient-derived breast cancer tissue assays may help to investigate different viruses, compare the effects of engineering tumor specificity or arming for enhanced oncolytic effects and select the optimal subtypes of breast cancer suitable for virotherapy (94). Such tissue assays would ideally include immune cells within the tumor environment to investigate interactions with the immune system or other immunotherapies. This information will allow clinicians to prioritize the testing of the most promising oncolytic viruses, investigate the best routes of administration, and choose the most effective agents to combine with oncolytic viruses in breast cancer treatment.

## Author Contributions

Conceptualisation: MC, AK, AH, and UL. Methodology: MC, AK, AH, and UL. Data Curation: MC. Writing: Original draft: MC Writing: Review & Editing: MC, AK, AH, and UL. Supervision: AK, AH, and UL. All authors contributed to the article and approved the submitted version.

## Funding

MC received bursary in the form of financial support from the Interdisciplinary Centre for Clinical Research (IZKF) of the Medical Faculty of the University of Tuebingen.

## Conflict of Interest

The authors declare that the research was conducted in the absence of any commercial or financial relationships that could be construed as a potential conflict of interest.

## Publisher’s Note

All claims expressed in this article are solely those of the authors and do not necessarily represent those of their affiliated organizations, or those of the publisher, the editors and the reviewers. Any product that may be evaluated in this article, or claim that may be made by its manufacturer, is not guaranteed or endorsed by the publisher.
