# Supplementary material for: Clinical Trials of Oncolytic Viruses in Breast Cancer
Source: Front Oncol. 2021 Dec 23;11:803050. doi: 10.3389/fonc.2021.803050 (PMC8733599; doi:10.3389/fonc.2021.803050)
Supplement: Supplementary file 1 [file Table_1.docx]

**S1. Published monotherapy and Combination studies with oncolytic viruses in breast cancer / solid tumors**

| Virus | Name | Mechanism | Result | Reference (DOI) |
| --- | --- | --- | --- | --- |
| Vaccinia virus | JX-929 or vvDD | Engineered for tumor selectivity through two targeted gene deletions. vvDD-CDSR was constructed using homologous recombination of the cytosine deaminase and somatostatin receptor genes into the TK-locus of VSC20 (a VGF deleted WR strain of VV) | Phase I, intratumoral dose escalation clinical trial in 16 patients with advanced solid tumors including breast. Intratumoral injection of the oncolytic vaccinia vvDD was well-tolerated in patients and resulted in selective infection of injected and noninjected tumors and antitumor activity. | 10.1038/mt.2014.194 |
| Reovirus | Pelareorep (Reolysin®) | Reolysin® is a purified live replication-competent form of the reovirus serotype 3 Dearing strain. REOvirus (Respiratory Enteric Orphan). It induces cytopathic and anti-cancer effects in cells with an activated ras pathway due to inhibition of the dsRNA-activated protein kinase. | Phase I study: 18 patients received 27 doses of Reolysin in 6 dose cohorts accomplishing a 300-fold dose escalation without a protocol-defined dose limiting toxicity. Drug related grade 2 toxicities included fatigue and fever (1 patient each). All patients developed neutralizing antibody during the course of the study. Viral shedding was observed in 6 patients. One patient with anthracycline and taxane refractory breast cancer experienced a partial response (PR) and her tumor had a ras G12A mutation. Biopsy from her chest wall mass showed evidence of necrosis and viral replication by electron microscopy. Overall clinical benefit (1 PR + 7 stable disease) rate was 45%, and appeared higher in patients with viral shedding (67%) than those without (33%)  Phase I study: 19 patients with various advanced solid tumors were treated. The most common toxicities related to treatment were grade 2 (or less) local erythema and transient flu like symptoms. Viral shedding was not seen in cerebral spinal fluid (CSF), urine and stool samples in all patients. Rising viral antibody titres were seen in all patients. In addition, we observed some evidence of local target tumor response activity in 7/19 patients (37 %) at the end of six or more weeks follow-up, with one patient exhibiting a complete response (CR), two a partial response (PR), and four stable disease (SD) to the local injected lesion. Conclusions Reolysin® is well tolerated given intralesionaly, with DLT/MTD not reached at a dose of 1010 PFU. The favorable toxicity profile, lack of viral shedding and possible therapeutic activity has made this unattenuated oncolytic reovirus an attractive cancer therapeutic agent for ongoing clinical studies, including in the setting of locally advanced accessible disease for palliation of symptoms.  Multicentre, randomized, phase II trial to evaluate the efficacy and safety of adding pelareorep to paclitaxel for patients with metastatic breast cancer (mBC).Following a safety run-in of 7 patients, 74 women with previously treated mBC were randomized either to paclitaxel 80 mg/m^2^ intravenously on days 1, 8, and 15 every 4 weeks plus pelareorep 3 × 1010 TCID50 intravenously on days 1, 2, 8, 9, 15, and 16 every 4 weeks (Arm A) or to paclitaxel alone (Arm B). Primary endpoint was progression-free survival (PFS). Secondary endpoints were objective response rate, overall survival (OS), circulating tumor cell counts, safety, and exploratory correlative analyses. Final analysis was performed after a median follow-up of 29.5 months. Pelareorep was well tolerated. Patients in Arm A had more favourable baseline prognostic variables. Median adjusted PFS (Arm A vs B) was 3.78 mo vs 3.38 mo (HR 1.04, 80% CI 0.76–1.43, P = 0.87). There was no difference in response rate between arms (P = 0.87). Median OS (Arm A vs B) was 17.4 mo vs 10.4 mo (HR 0.65, 80% CI 0.46–0.91, P = 0.1). This first, phase II, randomized study of pelareorep and paclitaxel in previously treated mBC did not show a difference in PFS (the primary endpoint) or RR. However, there was a significantly longer OS for the combination. | 10.1007/s10637-009-9279-8  10.1007/s10637-012-9865-z  10.1007/s10549-017-4538-4 |
| Adeno-virus | Ad5/3-24-GMCSF (CGTG-102) | Modified oncolytic adenovirus expressing granulocyte-macrophage colony-stimulating factor (GM-CSF) | Following serial treatment, both increase and decrease in antitumor T cells in blood were seen more frequently, findings which are compatible with induction of T-cell immunity and trafficking of T cells to tumors, respectively. Safety was good in both groups. In 115 patients treated with CGTG-102 (Ad5/3-D24-GMCSF), median overall survival was 111 days following single and 277 days after serial treatment in nonrandomized comparison. Switching the virus capsid for avoiding neutralizing antibodies in a serial treatment featuring three different viruses did not impact safety or efficacy. A correlation between antiviral and antitumor T cells was seen (P = 0.001), suggesting that viral oncolysis can result in epitope spreading and breaking of tumor-associated immunologic tolerance. Alternatively, some patients may be more susceptible to induction of T-cell immunity and/or trafficking.  Low-dose CP increased the efficacy of Ad5/3-D24-GMCSF in vitro and in a TNBC mouse model. In ATAP, treatments appeared safe and well-tolerated. Thirteen out of 16 breast cancer patients treated were evaluable for possible benefits with modified RECIST 1.1 criteria: 1 patient had a minor response, 2 had stable disease (SD), and 10 had progressive disease (PD). One patient is alive at 1,771 d after treatment. Ad5/3-D24-GMCSF in combination with low-dose CP showed promising efficacy in preclinical studies and possible antitumor activity in breast cancer patients refractory to other forms of therapy. | 10.1158/1078-0432  10.1080/2162402X.2015.1078057 |
| Adeno­virus | Ad5/3-E2F-Δ24-GMCSF (CGTG-602) | Quadruple modified oncolytic adenovirus expressing granulocyte-macrophage colony-stimulating factor (GM-CSF). Ad5/3-E2F-Δ24-GMCSF (CGTG-602) was engineered to contain a tumor specific E2F1 promoter driving an E1 gene deleted at the retinoblastoma protein binding site (“Δ24”). The fiber features a knob from serotype 3 for enhanced gene delivery to tumor cells. | Signs of antitumor efficacy were seen in 9/12 evaluable patients (75%). The radiological disease control rate with PET was 83% while the response rate was 50%. Tumor biopsies indicated accumulation of immunological cells, especially T-cells, to tumors after treatment. RNA expression analyses of tumors indicated immunological activation and metabolic changes secondary to virus replication. (phase II) | 10.18632/oncotarget.2901 |
| Adeno-virus | AD5-D24-GMCSF | Oncolytic adenovirus coding for GMCSF | Phase I trial: 20 patients with advanced solid tumors refractory to standard therapies were treated with Ad5-D24-GMCSF. Of the 16 radiologically evaluable patients, 2 had complete responses, 1 had a minor response, and 5 had disease stabilization. Responses were frequently seen in injected and noninjected tumors. Treatment was well tolerated and resulted in the induction of both tumor-specific and virus-specific immunity as measured by ELISPOT and pentamer analysis.  Patients with advanced solid tumors refractory to and progressing after conventional therapies were treated with three different regimens of low-dose cyclophosphamide (CP) in combination with oncolytic adenovirus. CP was given with oral metronomic dosing (50 mg/day, N = 21), intravenously (single 1,000 mg dose, N = 7) or both (N = 7). Virus was injected intratumorally. Controls (N = 8) received virus without CP. Treatments were well tolerated and safe regardless of schedule. Antibody formation and virus replication were not affected by CP. Metronomic CP (oral and oral + intravenous schedules) decreased regulatory T cells (Tregs) without compromising induction of antitumor or antiviral T-cell responses. Oncolytic adenovirus given together with metronomic CP increased cytotoxic T cells and induced Th1 type immunity on a systemic level in most patients. All CP regimens resulted in higher rates of disease control than virus only (all P < 0.0001) and the best progression-free (PFS) and overall survival (OS) was seen in the oral + intravenous group. One year PFS and OS were 53 and 42% (P = 0.0016 and P < 0.02 versus virus only), respectively, both which are unusually high for chemotherapy refractory patients. | 10.1158/0008-5472.Can-09-3567  10.1038/mt.2011.113 |
| Adeno­virus | Ad5-RGD-D24-GMCSF | p16/Rb pathway selective oncolytic adenovirus featuring RGD-4C modification of the fiber. This allows viral entry through alpha-v-beta integrins frequently highly expressed in advanced tumors. Granulocyte-macrophage colony stimulating factor (GM-CSF) is a potent activator of immune system with established antitumor properties. Ad5-RGD-D24-GMCSF features GM-CSF controlled by the adenoviral E3 promoter to stimulate antitumor immunity and break tumor-associated immunotolerance. | Treatments with Ad5-D24-RGD (N 5 9) and Ad5-RGD-D24-GMCSF (N 5 7) were well tolerated. Typical side effects were grade 1-2 fatigue, fever and injection site pain. 77% (10/13) of evaluable patients showed virus in circulation for at least 2 weeks. In 3 out of 6 evaluable patients, disease previously progressing stabilized after a single treatment with Ad5-RGD-D24- GM-CSF. In addition, 2/3 patients had stabilization or reduction in tumor marker levels. All patients treated with Ad5-D24-RGD showed disease progression in radiological analysis, although 3/6 had temporary reduction or stabilization of marker levels. Induction of tumor and adenovirus specific immunity was demonstrated with ELISPOT in Ad5-RGD-D24-GMCSF treated patients. (phase II) | 10.1002/ijc.26216 |
| Adeno­virus | ICOVIR-7 | ICOVIR-7 features an RGD-4C modification of the fiber HI-loop of serotype 5 adenovirus for enhanced entry into tumor cells. Tumor selectivity is mediated by an insulator, a modified E2F promoter, and a Rb-binding site deletion of E1A, whereas replication is optimized with E2F binding hairpins and a Kozak sequence. | ICOVIR-7 treatment was well tolerated with mild to moderate fever, fatigue, elevated liver transaminases, chills, and hyponatremia. One patient had grade 3 anemia but no other serious side effects. At baseline, 9 of 21 of patients had neutralizing antibody titers against the ICOVIR-7 capsid. Treatment resulted in neutralizing antibody titer induction within 4 weeks in 16 of 18 patients. No elevations of serum proinflammatory cytokine levels were detected. Viral genomes were detected in the circulation in 18 of 21 of patients after injection and 7 of 15 of the samples were positive 2 to 4 weeks later suggesting viral replication. Objective evidence of antitumor activity was seen in 9 of 17 evaluable patients. In radiological analyses, 5 of 12 evaluable patients had stabilization or reduction in tumor size. These consisted of one partial response, two minor responses and two cases of stable disease, all occurring in patients who had progressive disease before treatment. (phase II) | 10.1158/1078-0432.CCR-09-3167 |
| Adeno­virus | Telomelysin | Telomelysin is a novel, replication-competent adenovirus serotype 5-based adenoviral construct that incorporates a human telomerase reverse transcriptase gene (hTERT) promoter. hTERT encodes for the catalytic protein subunit of telomerase, a polymerase that acts to stabilize telomere lengths and is highly expressed in tumors but not in normal, differentiated adult cells.4,5. Additional modifications of Telomelysin include the replacement of the normal transcriptional element of viral E1B gene by an IRES (Internal Ribosomal Entry Site) sequence to minimize “leakiness” further enhancing specificity. | A phase I clinical trial was conducted in patients with advanced solid tumors. A single intratumoral injection (IT) of Telomelysin was administered to three cohorts of patients. Safety, response and pharmacodynamics were evaluated. 16 patients with a variety of solid tumors were enrolled. IT of Telomelysin was well tolerated at all dose levels. Common grade 1 and 2 toxicities included injection site reactions (pain, induration) and systemic reactions (fever, chills). hTERT expression was demonstrated at biopsy in 9 of 12 patients. Viral DNA was transiently detected in plasma in 13 of 16 patients. Viral DNA was detectable in 4 patients in plasma or sputum at day 7 and 14 post-treatment despite below detectable levels at 24 h, thereby suggesting viral replication. One patient had a partial response of the injected malignant lesion. Seven patients fulfilled Response Evaluation Criteria in Solid Tumors (RECIST) definition for stable disease at day 56 after treatment. | 10.1038/mt.2009.262 |
| Adeno­virus | H103 | Recombinant oncolytic type 2 adenovirus overexpressing the heat shock protein (HSP)70 protein inhibits primary and metastatic tumors through enhanced oncolytic activity and HSP-mediated immune responses against shared and mutated tumor antigens. | A phase I clinical trial of intratumoral injection of H103 was conducted in the patients with advanced solid tumors. A total of 27 patients were injected intratumorally with H103 in a dose-escalation study from a dose of 2.5 × 107 to 3.0 × 1012 viral particles (VPs). The maximum tolerated dose of H103 was not defined. Two patients developed dose-limiting toxicities of grade III fever at the dose of 1.5 × 1012 VP and transient grade IV thrombocytopenia at the dose of 3.0 × 1012 VP. The common adverse events were mainly mild to moderate (grade I/II) in nature, including fever, mild injection-site reaction, leucopenia, lymphopenia, thrombocytopenia and hypochromia. The objective response (complete response + partial response) to H103-injected tumors was 11.1% (3/27), and the clinical benefit rate (complete response + partial response + minor response + stable disease) was 48.1%. Transient and partial regression of distant, uninjected tumors was observed in three patients. | 10.1038/gt.2008.179 |
| Adeno­virus | ONYX-015 ( CI-1042,dl-1520, lontucirev | ONYX-015 is a chimeric human group C adenovirus (Ad2 and Ad5) that does not express the 55 kDa product of the E1B gene. The virus contains a deletion between nucleotides 1496 and 3323 in the E1B region encoding the 55 kDa protein. In addition, a C to T transition at position 2022 in E1B generates a stop codon at the third codon position of the protein. These alterations eliminate expression of the E1B 55 kDa gene in ONYX-015-infected cells. | Phase I, dose-escalation study to determine the safety and feasibility of intravenous infusion with ONYX-015 in combination with Enbrel in patients with advanced carcinoma. Nine patients, three in each cohort received multiple cycles of ONYX-015 infusion (1 × 1010, 1 × 1011 and 1 × 1012 vp weekly for 4 weeks/cycle) in addition to subcutaneous enbrel (only during cycle 1) injections per FDA-indicated dosing. Of the nine patients, four had stable disease. Two of the three patients in cohort 3 had detectable viral DNA at days 3 and 8 post-ONYX-015 infusion. Their detectable circulating viral DNA was markedly higher during cycle 1 (with enbrel coadministration) as compared with cycle 2 (without enbrel) at the same time points. Area under the curve determinations indicate a marked higher level of TNF-α induction and accelerated clearance at cycle 2 in the absence of enbrel. | 10.1038/sj.cgt.7701080 |
| Newcastle disease virus | PV701 | PV701 is a highly purified, naturally attenuated, replication competent isolate of Newcastle disease virus, an avian paramyxovirus that is not a human pathogen. This oncolytic virus directly lyses the vast majority of diverse human cancer cell lines tested in vitro. Most tumor cells are at least 1,000-fold more sensitive to PV701 cytolysis than normal cells. PV701 is selectively cytotoxic, exploiting defects in IFN antiviral responses common in tumor cells | Phase I: A 100-fold dose intensification was  achieved over 195 cycles. A first-dose MTD of 12 x 10^9^ plaque-forming units (PFU)/m^2^ was established for outpatient dosing. After an initial dose of 12 x 10^9^ PFU/m^2^, patients tolerated an MTD for subsequent doses of 120 x 10^9^ PFU/m2. The most common adverse events were flu-like symptoms that occurred principally after the first dose and were decreased in number and severity with each subsequent dose. Tumor site–specific adverse events and acute dosing reactions were also observed but not cumulative toxicity. Objective responses occurred at higher dose levels, and progression-free survival ranged from 4 to 31 months. Tumor tissue from one patient was obtained after 11 months of therapy and showed evidence of PV701 particles budding from the tumor cell membrane by electron microscopy and a pronounced lymphoplasmacytic infiltrate by histologic examination.  Phase I study tested the hypothesis that two-step desensitization, using two dose increments before high repeat doses, would be well tolerated. Sixteen adults with incurable solid tumors were enrolled. Cycles consisted of six PV701 doses over 2 weeks followed by a 1-week rest. Doses 1 to 2 were 1 and 12 × 109 plaque-forming units (pfu)/m2, respectively, whereas doses 3 to 6 were escalated by cohort from 24 to 120 × 109 pfu/m2. No dose-limiting toxicities were observed, permitting dose escalation through cohort 4 (1, 12, 120, 120, 120, 120 × 109 pfu/m2). Mild flu-like symptoms were common following the first infusion, diminished with repeated dosing, and were less pronounced than those seen previously. Tumor regression was observed in a patient with anal carcinoma who enrolled with stable disease following palliative radiotherapy. Four patients with clearly progressing cancer before enrollment had disease stabilization of ≥6 months. | 10.1200/JCO.2002.08.042  10.1158/1078-0432 |
| Herpes simplex virus  type 1 | HF10 | An oncolytic herpes simplex virus type 1, mutant HF10, has been isolated and evaluated for anti-tumor efficacy in syngeneic immunocompetent mouse models. The mutant virus can have potential to treat cancer in experimental studies using animals, and that all of the surviving mice acquire resistance to re-challenge of the tumor cells. | Phase I studies using HF10 were initiated in patients with metastatic breast cancer. For each patient, 0.5 ml HF10 diluents at various doses were injected into test nodule, and 0.5 ml sterile saline was injected into a second nodule. While no adverse effects occurred, there was cancer cell death and 30-100% regression histopathologically in recurrent breast cancer. A histological examination revealed fibrosis and tumor cell death with an infiltration of CD8+ and CD4+ T-cells around tumor islets, findings that support the induction of an immune response by HF10 | 10.2174/156800907780058808  10.1245/ASO.2006.08.035  10.1038/cgt.2011.80 |
| Herpes simplex virus  type 1 | OncoVEX^GM-CSF^ | This virus was developed to induce a more potent antitumor immune response through the deletion of the HSV gene encoding ICP47, which blocks antigen presentation in HSV-infected cells and the delivery of the gene encoding human granulocyte macrophage colony-stimulating factor (GM-CSF). Improved oncolysis was achieved through the use of a more potent clinical isolate of HSV for construction of the virus, and, in addition to the deletion of ICP34.5, which is well documented to provide tumor-selective virus replication, the expression of the US11 gene as an immediate early rather than late gene that has previously been shown to boost tumor-selective virus replication | The virus was administered by intratumoral injection in patients with cutaneous or s.c. deposits of breast, head and neck and gastrointestinal cancers, and malignant melanoma who had failed prior therapy. Thirteen patients were in a single-dose group, where doses of 10^6^, 10^7^, and 10^8^ plaque-forming units (pfu)/mL were tested, and 17 patients were in a multidose group testing a number of dose regimens. The virus was generally well tolerated with local inflammation, erythema, and febrile responses being the main side effects. The local reaction to injection was dose limiting in HSV-seronegative patients at 10^7^ pfu/mL. The multidosing phase thus tested seroconverting HSV-seronegative patients with 10^6^ pfu/mL followed by multiple higher doses (up to 10^8^ pfu/mL), which was well tolerated by all patients. Biological activity (virus replication, local reactions, granulocyte macrophage colony-stimulating factor expression, and HSV antigen-associated tumor necrosis), was observed. The duration of local reactions and virus replication suggested that dosing every 2 to 3 weeks was appropriate. Nineteen of 26 patient post-treatment biopsies contained residual tumor of which 14 showed tumor necrosis, which in some cases was extensive, or apoptosis. In all cases, areas of necrosis also strongly stained for HSV. The overall responses to treatment were that three patients had stable disease, six patients had tumors flattened (injected and/or uninjected lesions), and four patients showed inflammation of uninjected as well as the injected tumor, which, in nearly all cases, became inflamed. | 10.1158/1078-0432.CCR-06-0759 |

**S2. Unpublished clinical studies with oncolytic viruses in breast cancer**

| Virus | Name(s) | Phase | Mechanism | Study | Status | Trialtrove ID |
| --- | --- | --- | --- | --- | --- | --- |
| Adeno­virus | Ad3-hTERT-E1A | I | Fully serotype 3 oncolytic adenovirus. The E1A region of the virus is controlled by an hTERT promoter | Dose-escalation was ongoing in a clinical trial in 8 patients with advanced solid tumors refractory to standard therapies. Preliminary results showed that Ad3-hTERT-E1A was safe. All patients experienced grade 1-2 adverse events, typically fever, nausea and tiredness. No grade 3 or higher adverse events were observed | Completed/  development discontinued | TrialTroveID-126529 |
|  | Ad5/3-D24-RGD (ONCOS-102, CGTG-102) | I | CGTG-102 is an oncolytic adenovirus utilizing a defect in the Rb-p16 pathway. It is a genetically modified serotype 5/3 adenovirus coding for granulocyte-macrophage colony-stimulating factor. Engineered human serotype 5 adenovirus optimized to induce systemic anti-tumor T cell response | Serial treatment of cancer patients with GM-CSF expressing oncolytic adenovirus enhances immunological response and survival without compromising safety | Completed | TrialTroveID-156317 |
|  | DWP-418 | I | Contains a modified hTERT promoter, resulting in virus activation in cancer cells expressing high telomerase levels, and expresses a relaxin gene to increase viral spreading efficiency and stimulate apoptosis | Dose-escalation, cohort study in patients with head & neck cancer and melanoma cancer. Primary Endpoints: DLT (Dose limiting toxicity), MTD (Maximum tolerable dose). Secondary Endpoints: evaluate clinical and biologic anti-tumor effect. | Completed/  development discontinued | TrialTroveID-105429 |
|  | Telomelysin, OBP-301 | I | Oncolytic adenovirus derived from human adenovirus type 5, in which the normal transcriptional element of the E1A gene has been replaced by a human telomerase reverse transcriptase (hTERT) promoter sequence | Intratumoral injection with telomerase-specific replication-competent oncolytic adenovirus, telomelysin (OBP-301) for the treatment of various solid tumors to determine the feasibility, safety and to characterize the pharmacokinetics in patients with advanced solid tumors. | Completed | TrialTroveID-056832 |
|  | enadenotucirev | I/II | Selective Ad3/Ad11p chimeric virus and is the 1st described non-Ad5 oncolytic adenovirus | Multicentre observational study for the long-term follow-up of subjects that have been treated with enadenotucirev during Interventional clinical trial  A clinical study of enadenotucirev administered by sub-acute fractionated intravenous injection: dose escalation In metastatic epithelial solid tumors and randomised controlled trial In metastatic colorectal cancer EValuating OncoLytic Vaccine Efficacy (Evolve). | Planned  Completed | TrialTroveID-319265  TrialTroveID-170847 |
|  | H-101, oncorine | II | E1B-55kd deleted segment in the virus, which results in the ability to replicate and lyse tumor cells selectively, whilst leaving normal cells unaffected. | To evaluate anti-tumor activity and toxicity of H101 in combination with chemotherapy in patients with late stage cancers and to test the effect of H101 on a wide type of advanced cancers. | Completed | TrialTroveID-051907 |
|  | lontucirev, ONYX-015 | I | ONYX-015 is a chimeric human group C adenovirus (Ad2 and Ad5) that does not express the 55 kDa product of the E1B gene. The virus contains a deletion between nucleotides 1496 and 3323 in the E1B region encoding the 55 kDa protein. In addition, a C to T transition at position 2022 in E1B generates a stop codon at the third codon position of the protein. These alterations eliminate expression of the E1B 55 kDa gene. | Define the maximum tolerated dose (amount of product that can be given without producing severe side effects) with the fewest side effects of ONYX-015 in combination with Enbrel when given in multiple infusions for the treatment of advanced cancer and determine the safety and feasibility of intravenous infusion with ONYX-015 in combination with enbrel in patients with advanced carcinoma. | Completed/  development discontinued | TrialTroveID-049815 |
|  | MAGE-A3,  Ad MAGEA3 | I/II | Adenovirus vaccine expressing MAGE-A3 antigen | Study of MG1 Maraba/MAGE-A3 (MG1MA3), with and without adenovirus vaccine, with transgenic MAGE-A3 insertion (AdMA3) in patients with Incurable advanced/metastatic MAGE-A3-expressing solid tumors | Closed | TrialTroveID-181181 |
|  | CGT-A310 | I | Tumor necrosis factor-related apoptosis-inducing ligand (TRAIL) Ad5 E1A | An open label phase I study of CGT-A310, a tropism mediated oncolytic adenovirus, in patients with treatment-refractory metastatic tumors | Completed | TrialTroveID-077327 |
|  | Adenovirus-based HER2 vaccine | I/II | Genetically engineered adenoviral cancer vaccine with the unique biological signature of the patient`s tumor antigen (CEA) | A Phase I/II Study of haNK in Combination with Trastuzumab (Herceptin) and an Adenovirus-based HER2 Vaccine in Patients with Breast Cancer | Planned | TrialTroveID-281678 |
|  | DNX-2440 | I | OX40-ligand expression | A phase I trial and window-of-opportunity Study of Preoperative Intratumoral Injection of OX-40 ligand Expressing oncolytic Adenovirus (DNX-2440) in patients with resectable lives metastases | Open | TrialTroveID-394732 |
|  | VISTA | I | VISTA (VIrus Specific T Cells and Adenovirus) | VISTA (VIrus Specific T Cells and Adenovirus): A First in Human Phase I Trial of Binary Oncolytic Adenovirus in Combination With HER2-Specific CAR VST Cells in Patients With Advanced HER2 Positive Solid Tumors | Planned | TrialTroveID-336851  NCT03740256 |

| Virus | Name(s) | Phase | Mechanism | Study | Status | Trialtrove ID |
| --- | --- | --- | --- | --- | --- | --- |
| Vaccinia virus | GL-ONC1 | I | Genetically-stable oncolytic vaccinia virus | Study of intra-pleural administration of GL-ONC1, a genetically modified Vaccinia virus, in patients with malignant pleural effusion: primary, metastases and mesothelioma | Closed | TrialTroveID-161382, NCT01766739 |
|  | JX-929,  vvDD-CDSR | I | Armed oncolytic virus targeting the EGFR and RAS signal transduction pathways based on a modified vaccinia virus with a single deletion in a gene required for replication in normal cells, and replicates selectively in cancer cells. In addition, it has an inserted cytosine deaminase gene which stimulates anticancer immunity locally and against distant metastases | Dose-escalation trial Of Vvdd-CDSR (double-deleted vaccinia virus plus CD/ SMR) administered by intratumoral injection or intravenous injection | Completed | TrialTroveID-081339,  NCT0057497 |
|  | Pexastimogene devacirepvec, JX-594 | I/II | Armed oncolytic virus targeting the E2F based on a modified vaccinia virus with a single deletion in the thymidine kinase gene required to enhance replication in normal cells, and replicates selectively in cancer cells. In addition, it has an inserted GM-CSF transgene, which stimulates anticancer immunity locally and against distant metastases | A phase I dose-escalation trial evaluating the impact of an in Situ immunization strategy with intratumoral injections of Pexa-Vec in combination with ipilimumab in metastatic / advanced solid tumors with injectable lesions  A phase I/II study of Pexa-Vec in combination with metronomic cyclophosphamide in patients with advanced solid tumors  A phase I/II study of metronomic cyclophosphamide and oncolytic poxvirus JX-594 in patients with advanced breast cancer and advanced soft tissue sarcoma | Open  Open  Terminated, lack of efficacy | TrialTroveID-267839, NCT02977156  TrialTroveID-203581  TrialTroveID-269815  NCT02630368 |
|  | BT-001 | I/IIa | Vaccinia virus expressing anti-CTLA4 antibody and GM-CSF | A phase I/IIa study of intra-tumoral BT-001 (TG6030) administered alone and in combination with Pembrolizumab in patients with cutaneous or, subcutaneous lesions or easily injectable lymph nodes of metastatic/advanced solid tumors | Open | TrialTroveID-335302 |
|  | TBio-6517 | I/IIa | Vaccinia virus expressing three potent immune modulators | A phase I/II, multicenter, open-label trial of TBio-6517, an oncolytic vaccinia virus, administered by intratumoral injection, alone and in combination with Pembrolizumab, in patients with advanced solid tumors | Open | TrialTroveID-369342 |
|  | CF33 | I/II | Chimaeric vaccinia oncolytic virus | A Phase I Study Of CF33 In Patients With Advanced Solid Tumors | Planned | TrialTroveID-353596 |

| Virus | Name(s) | Phase | Mechanism | Study | Status | Trialtrove ID |
| --- | --- | --- | --- | --- | --- | --- |
| Herpes simplex virus | Msc-2a,  HF10, TBI-1401 | I | Attenuated strain of the herpes simplex virus 1 (HSV-1) | Phase I trial of intratumoral administration of HF10, a replication competent Herpes Simplex Virus Type 1, in patients with refractory head and neck cancer or solid tumors with cutaneous and/or superficial lesions  Pilot phase I study of oncolytic viral therapy using mutant Herpes Simplex Virus (HF10) against recurrent metastatic breast cancer | Completed  Completed | TrialTroveID-119180  NCT01017185  TrialTroveID-098182 |
|  | RP-1 | I/II | Herpes simplex virus expressing fusogenic GALV-GP R- protein and GM-CSF | An open-label, multicenter, phase I/II of RP-1 as single agent and in combination with PD1 blockade in patients with solid tumors | Open | TrialTroveID-314671 |
|  | RP-2 | II | RP-2 is derived from RP-1 which expresses an anti-CTLA-4 antibody-like molecule inhibiting T-cell activation | Phase II study of RP2 with Anti-PD-1 in triple negative breast cancer | Planned | TrialTroveID-341369 |
|  | recombinant human GM-CSF type II herpes simplex virus vaccine | I  I/II  I/II | OH2 is a recombinant human GM-CSF oncolytic virus, developed upon genetic modifications of the herpes simplex virus type 2 strain HG52, allowing the virus to selectively replicate in tumors | Phase I Clinical Trial of Oncolytic Virus OH2 in the Treatment of Advanced Solid Tumors  Open and Incremental Phase I Clinical Trial of Recombinant Human GM-CSF Type II Herpes Simplex Virus (OH2) Injection (Vero Cells) in the Treatment of Advanced Solid Tumors  Phase I/II Study of OH2 Injection, an Oncolytic Type 2 Herpes Simplex Virus Expressing Granulocyte Macrophage Colony-Stimulating Factor, in Malignant Solid Tumors | Open  Open  Open | TrialTroveID-340707  TrialTroveID-374423  NCT04386967  TrialTroveID-344776  NCT03866525 |
|  | ONCR-177 | I | ONCR-177 is a next generation oncolytic herpes simplex virus (HSV) therapeutic targeting IL-12, FLT3L, CCL4, CTLA-4 and PD-1 | A Phase I, Open-Label, Multicenter, Dose Escalation and Expansion Study of ONCR-177, an Oncolytic Herpes Simplex Virus for Intratumoral Injection, Alone and in Combination With PD-1 Blockade in Adult Subjects With Advanced and/or Refractory Cutaneous, Subcutaneous or Metastatic Nodal Solid Tumors | Open | TrialTroveID-337871  NCT04348916 |
|  | Talimogene laherparepvec (OncoVEX^GM-CSF^) | I/II | Armed oncolytic immunotherapy based on OncoVEX^GM-CSF^, an oncolytic herpes simplex virus-1 derivative, which contains a single mutation rendering it non-pathogenic to non-tumor cells and upregulating oncolytic properties. It also expresses granulocyte-macrophage colony stimulating factor (GM-CSF) | A phase II study using talimogene laherparepvec as a single agent for inflammatory breast cancer (IBC) or non-IBC patients with inoperable local recurrence  A first-in-human, 2-part, open label pharmacokinetic phase I/II clinical trial of OncoVEX^GM-CSF^ in patients with solid tumors  A phase Ib/II, multicenter, open-label trial to evaluate the safety of talimogene laherparepvec injected into liver tumors alone and in combination with systemic pembrolizumab  A phase I/II study of talimogene laherparepvec in combination with neoadjuvant chemotherapy in triple negative breast cancer  A phase Ib study of talimogene laherparepvec in combination With atezolizumab in subjects with triple negative breast cancer and colorectal cancer with liver metastases  A phase I, multicenter, open-label, dose de-escalation study to evaluate the safety and efficacy of talimogene laherparepvec in paediatric subjects with advanced non central nervous system tumors amenable to direct injection  A phase 1b study of talimogene laherparepvec (T-VEC) in combination with paclitaxel or endocrine therapy in patients with metastatic, unresectable, or locoregionally recurrent HER2-negativebBreast cancer with evidence of injectable disease in the locoregional area  Combination of Talimogene Laherparepvec With Atezolizumab in Patients With Residual Breast Cancer After Standard Neoadjuvant Multi-agent Chemotherapy (PROMETEO TRIAL)  A Phase I Study of Ipilimumab, Nivolumab and Talimogene Laherparepvec Preoperative Treatment of Localized Breast Cancer-deleted | Closed  Completed  Open  Closed  Closed  Open  Open  Open  Closed | TrialTroveID-271702  NCT02658812  TrialTroveID-010397  TrialTroveID-262177  NCT02509507  TrialTroveID-279209  NCT02779855  TrialTroveID-258657  NCT03256344  TrialTroveID-276285  NCT02756845  TrialTroveID-326015  NCT03554044  TrialTroveID-340773  NCT03802604  TrialTroveID-362684 |

| Virus | Name(s) | Phase | Mechanism | Study | Status | Trialtrove ID |
| --- | --- | --- | --- | --- | --- | --- |
| Reovirus | pelareorep (Reolysin) | I/II/III | Naturally occurring oncolytic reovirus | A phase I study of the combination of intravenous reovirus type 3 Dearing and gemcitabine in patients with advanced cancer  A phase I clinical trial of terminal cancer patients with progressive (actively growing) cancers that had failed to respond to conventional treatments was performed  An open-label, single-arm, multi-centre phase II study of Reolysin delivered via intratumoral injection with low-dose radiotherapy in patients with advanced cancer  Phase I clinical trial of systemic delivery of Reolysin as a treatment for patients with advanced or metastatic solid tumors  A phase I clinical trial using intravenous administration of REOLYSIN in combination with docetaxel (Taxotere) in patients with advanced cancers  A Multi-Center, Single-Arm, Open Phase I Clinical Trial To Evaluate The Safety, Tolerability, And Pelareorep In Vivo Process Of Pelareorep Combined With Paclitaxel Injection In Chinese Patients With Advanced Or Metastatic Breast Cancer  A randomized phase II study of Reolysin for patients receiving standard weekly paclitaxel therapy as therapy for advanced/metastatic breast cancer  A phase II partner-sponsored window of opportunity study of pelareorep in combination with standard of care therapy in the neoadjuvant Setting in metastatic breast cancer (mBC)  A pivotal phase III study to evaluate Reolysin in combination with paclitaxel in patients with HR Positive/HER2 negative metastatic breast cancer  A phase II partner-sponsored basket study to generate important biomarker and efficacy data of pelareorep in combination with checkpoint inhibitors  A Open-label, Randomized, Multicenter, Phase III Study of AN1004 in Combination with Paclitaxel in Patients with Advanced or Metastatic Breast Cancer  An Open Label Phase II Study Of Pelareorep In Combination With Paclitaxel And Avelumab In Patients with HR Positive/HER2 Negative Metastatic Breast Cancer BReast cAnCEr with the Oncolytic Reovirus PeLareorEp in CombinaTion with anti-PD-L1 and Paclitaxel - BRACELET-1  A Pivotal Phase III Study to Evaluate Reolysin in Combination with Paclitaxel in Patients with HR Positive/HER2 Negative Metastatic Breast Cancer (Phase III)  IRENE Study: Phase II Study of INCMGA00012 and the Oncolytic Virus Pelareorep in Metastatic Triple Negative Breast Cancer | Completed  Completed  Completed  Completed  Completed  Planned  Completed  Open  Planned  Planned  Planned  Open  Planned  Open | TrialTroveID-062409  TrialTroveID-010034  TrialTroveID-053244  TrialTroveID-026816  TrialTroveID-062412  TrialTroveID-386449  TrialTroveID-169843  NCT01656538  TrialTroveID-322037  TrialTroveID-298624  TrialTroveID-322124  TrialTroveID-346197  TrialTroveID-351980  TrialTroveID-298624  TrialTroveID-377822 |

| Virus | Name(s) | Phase | Mechanism | Study | Status | Trialtrove ID |
| --- | --- | --- | --- | --- | --- | --- |
| Coxsackie group A virus | CVA-21 (Cavatak) | I | Oncolytic are attributable to their ability to bind to intercellular adhesion molecule 1 (ICAM-1) and decay-activating factor (DAF), overexpressed on certain cancer cells, and to subsequently induce apoptosis | A phase I, open-label, cohort study of multiple doses of Cavatak (Coxsackievirus A21) given intravenously to stage IV solid tumor cancer patients bearing ICAM-1 with or without DAF expressing tumors  A Phase Ib/II Clinical Study of Intratumoral Administration of V937 in Combination With Pembrolizumab (MK-3475) in Participants With Advanced/ Metastatic Solid Tumors | Completed  Open | TrialTroveID-085664  NCT00636558  TrialTroveID-382400  NCT04521621 |
| Polio virus vaccine | PVS-RIPO | I | PVSRIPO is the live attenuated, oral (Sabin) serotype 1 poliovirus vaccine containing a heterologous internal ribosomal entry site (IRES) derived from the human rhinovirus type 2 (HRV2) | Examining oncolytic Poliovirus bioactivity in tumor tissue after intratumoral administration of PVSRIPO in women With triple negative breast cancer | Open | TrialTroveID-326695  NCT03564782 |
| Measles virus | oncolytic measles virus/MV-NIS | I | Encoding thyroidal sodium iodide symporter | Phase I trial of intratumoral administration of a NIS-expressing derivative manufactured from a genetically engineered strain of Measles virus in patients with recurrent/metastatic squamous cell carcinoma of the head and neck or metastatic breast cancer | Closed | TrialTroveID-197116  NCT01846091 |
|  | MV-s-NAP | I | Measles Virus Derivative Expressing the Helicobacter Pylori Neutrophil-Activating Protein | Phase I Trial of Intratumoral Administration of a Measles Virus Derivative Expressing the Helico­bacter Pylori Neutrophil-Activating Protein (NAP) (MV-s-NAP) in Patients With Metastatic Breast Cancer | Open | NCT04521764  TrialTroveID-382561 |
| Newcastle disease virus | PV-701 | I | PV-701 is a replication-competent Newcastle disease virus strain | Phase I trial of intravenous administration of PV701, an oncolytic virus, in patients with advanced solid cancers  A phase 1 clinical study of intravenous administration of PV701, an oncolytic virus, using two-step desensitization | Completed  Completed | TrialTroveID-006839  TrialTroveID-144288 |
|  | MEDI-5395 | I | Genetically engineered NDV with the insertion of GMCSF | An open-label phase I study to assess the safety, tolerability, pharmacokinetics, pharmacodynamics and preliminary efficacy of MEDI-5395 with durvalumab in subjects with selected advanced solid tumors | Open | TrialTroveID-346121 |
| Vesicular stomatitis virus | Vyria, Voyager-V1, VSV IFNb NIS | I | Genetically engineered to express NIS and Human Interferon Beta (VSV-IFN-beta-NIS) | Phase I Trial of Vesicular Stomatitis Virus Genetically Engineered to Express NIS and Human Interferon Beta (VSV-IFNb-NIS) Monotherapy and in Combination With Avelumab, in Patients With Refractory Solid Tumors | Open | TrialTroveID-287824  NCT02923466 |
| Retroviral Replicating Vector | Toca-511 | I | Toca-511 (vocimagene amiretrorepvec) is a live virus which carries a cytosine deaminase gene intended to be used in combination with Toca FC, a novel extended-release tablet containing 5-FC (flucytosine).The cytosine deaminase carried by Toca-511 converts the prodrug 5-flucytosine (5-FC) into 5-fluorouracil (5-FU) | A phase Ib study of Toca 511, a retroviral replicating vector, combined with Toca FC in patients with solid tumors or lymphoma | Terminated | TrialTroveID-264073  NCT02576665 |
